# Case report of Chromosome 3q25 deletion syndrome or Mucopolysaccharidosis IIIB

**DOI:** 10.7603/s40681-014-0007-0

**Published:** 2014-08-06

**Authors:** Yu-Tzu Chang, Chung-Hsing Wang, I-Ching Chou, Wei-De Lin, Siew-Yin Chee, Huang-Tsung Kuo, Fuu-Jen Tsai

**Affiliations:** 1 Division of Pediatric Neurology, Children’s Medical Center, China Medical University Hospital, Taichung, Taiwan; 2 China Medical University, Taichung, Taiwan; 3 Division of Pediatric Genetics and Metabolism, Children’s Medical Center, Taichung, Taiwan; 4 Graduate Institute of Clinical Medical Science, China Medical University, Taichung, Taiwan; 5 Graduate Institute of Integrated Medicine, College of Chinese Medicine, China Medical University, Taichung, Taiwan; 6 Department of Medical Research, China Medical University Hospital, Taichung, Taiwan; 7 School of Post Baccalaureate Chinese Medicine, China Medical University, Taichung, Taiwan; 8 Division of Children’s Development and Behavior, Children’s Medical Center, China Medical University Hospital, Taichung, Taiwan; 9 School of Chinese Medicine, China Medical University, Taichung, Taiwan; 10 Department of Medical Genetics, China Medical University Hospital, Number 2, Yuh-Der Road, Taichung, Taiwan; 11 Department of Health and Nutrition Biotechnology, Asia University, Taichung, Taiwan

**Keywords:** Chromosome 3q deletion, Mucopolysaccharidosis

## Abstract

Interstitial deletions of the long arm of chromosome 3 have, to our knowledge, been reported in only eleven patients; detailed genotype- phenotype correlations are not well established. Here we describe a case with interstitial deletion involving 3q25.33 region. Dysmorphic features and developmental delay lead to clinical genetic and enzyme assessment. Low alpha-hexosaminidase level is also noted, which imply Mucopolysaccharidosis(MPS) IIIB.

## 1. Introduction

Interstitial deletions involving the long arm of chromosome 3 are rare and detailed genotype-phenotype correlations have not been well established to date. Proximal 3q deletion syndrome was delineated by Simovich et al. They concluded that these patients have a distinct recognizable facial dysmorphism and are at risk for developmental delay plus other structure abnormalities of the brain, genitor-urinary and musculoskeletal system. Furthermore, only 11 cases have been reported with distal deletions involving 3q25 regions. Among them, variable chromosomal breakpoints and deletion sizes located from 3q23 to 3q26.1 have been reported [1-11]. All patients presented facial dysmorphism and developmental delay, but other phenotypic features, such as cardiac defect, microcephaly, epicanthus and short stature were not always present.

We report a patient with interstitial deletion involving the 3q25.33 region and compare her phenotype with 11 previously reported cases. This report will add more specific clinical profiles to this unique disease spectrum.

### Methods and Results

#### 1.1 Clinical description

**Figure 1A&B:**
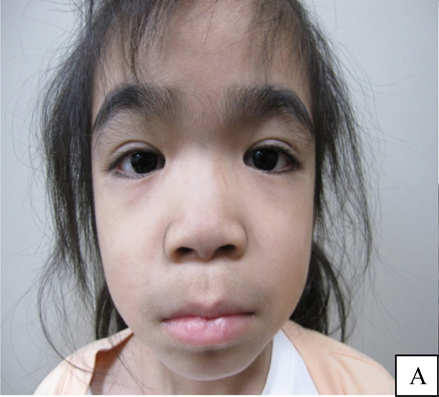

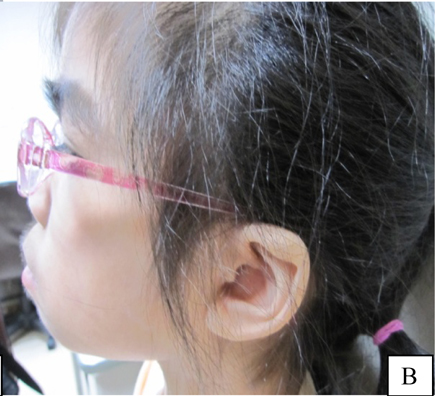
This 11 year-old girl with (A) coarse face, hirsutism, hypertelorism, synophrys, bilateral mild epicanthic folds, broad and flat nasal bridge. (B) low set, posterior rotated and mild dysplastic ear.

The eleven-year-old female patient was referred for evaluation of dysmorphic features and developmental delay at age eight. She was the first child of healthy and non-consanguineous parents; family history was not contributory. The pregnancy itself was not complicated, the child born by term vaginal delivery with no adverse perinatal events observed. Her birth body weight at birth was 3250 gm (50 percentile), her length 52 cm (85-97 percentile). She was noticeably overactive, talkative, easily distracted and emotionally labile since age three. Her school performance was poor. Facial abnormalities (Fig 1A&B) included a coarse face, hairy skin, synophrys, bilateral mild epicanthic folds, but no blepharophimosis. Nasal bridge was broad and flat, nasal tip bulbous in addition to having anteverted nostrils. Philtrum was smooth. Her mouth was observed as having a full lip and her palate intact. Ears were set low, with posterior rotation and mild dysplasy noted. She had normal palmer creases, no clinodactyly of fingers. She had facial hirsutism and hypertrichosis of limbs. All the patient’s cardiovascular, abdominal, genito-urinary and neurologic examinations were normal. Neurodevelopmental assessment by Wechsler Preschool and Primary Scale of Intelligence-Revision (WPPSI-R) was 60 and Adaptive Behavior Assessment System (ABAS) revealed mild adaptive disability. Attention deficit hyperactivity disorder (ADHD) was also diagnosed during assessment. In addition, enzyme analysis for mucopolysaccharidosis was performed due to coarse facial features and developmental delay. Low alpha-hexosaminidase level was found with 0.16 nmol/17h/mg (normal range 17.2±6.4 nmol/17h/mg) in the whole blood leukocyte. Therefore, mucopolysaccharidosis (MPS) type IIIB was also diagnosed. She started rehabilitation, along with behavioral and art therapies, for treatment of ADHD.

#### 1.2 Chromosome study

**Figure 2:**
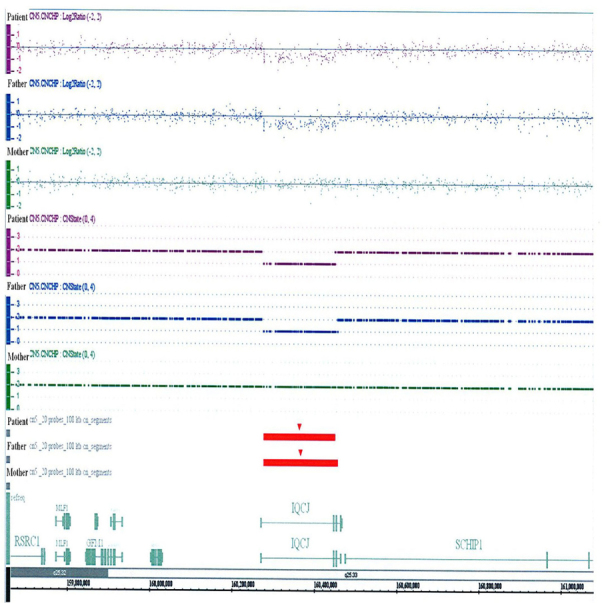
Genome-Wide Human SNP Array 6.0 was performed and revealed a deletion at cytoband 3q25.33; physical position 160.277-160.450 Mb about the size of 173 Kb. Her father had the same cytogenic deletion

Chromosome preparation was performed from peripheral white blood cells, and trypsin-banding Giemsa was applied 550 band resolution. G-banding analysis revealed that the patient had a karyotype of 46, xx, deletion at cytoband 3q25.33 (Fig 2).

### Discussion

Table 1 compares phenotype of our case with that of 11 previously reported 3q25 deletions [1-11]; female predominance was observed (10/12). Despite phenotypic variability, some clinical features, especially facial dysmorphism, were notable and comparable to cases reported earlier: e.g., synophrys, broad nasal bridge, large or abnormal ears. Developmental delay was also noted. Our patient had similar clinical facial features: synophrys, epicanthus, broad nasal bridge and ear abnormalities. Her parents also agreed to have genetic testing performed. Her mother’s genotype was normal. Her father had the same deletion point at 3q25.33 but with normal appearance, which may suggest variable expressivity in 3q25 deletion syndrome.

**Table 1.** Clinical features of this and 11 additional previously reported patients with interstitial deletion in 3q25

	Previous 11 cases (%) Present case	
Sex	M/F (2/9)	F
Parental karyotypes normal	10/10 (100%)	Paternal inheritance
Developmental delay	11/11 (100%)	+
Micorcephaly	5/10 (50%)	-
Synophrys	6/8 (75%)	+
Epicanthus	7/11 (64%)	+
Ptosis	7/11 (64%)	-
Blepharophimosis	6/11 (55%)	-
Broad nasal bridge	11/11 (100%)	+
Ear abnormalities	10/10 (100%)	+
Cardiac defect	5/8 ( 63%)	-

+, present; -, absent; M, male; F, female

Associated neuropsychiatric disorder was only mentioned in one case report of autistic and obsessive behavioral traits involved with 3q25 deletion [10, 12]. In our patient, ADHD was noted with adaptive disability. Significance of attention deficit traits in the context of this chromosome deletion in our patient is uncertain at this time. However, specific behavioral phenotypes (like obsessive behavior) are recognized features of chromosomal deletion syndromes [13-14].

Another issue noted in this case was low alpha-hexosaminidase level, indicating that her as a possible victim of mucopolysaccharidosis. Mucopolysaccharidosis-IIIB (MPS- IIIB) or Sanfilippo-B syndrome is caused by deficiency of lysosomal α-N-acetylglucosaminidase that leads to accumulation of heparan-sulphate and degeneration of the central nervous system with progressive dementia, hyperactivity, and aggressive behavior [15-16]. Clinical dysmorphic features of MPS III are less pronounced than those of other MPS types. Individuals with MPS III typically have mildly "coarse" facial features, a large head (macrocephaly), lower abdomen (inguinal hernia), restless behavior and vision problems, which also resemble our present case. However, there was no other skeletal abnormalities, chronic diarrhea, recurrent upper airway infections, sleep disorder, hearing loss, nor hepatomegaly. Despite developmental delay, there was no sign of regression observed in our case. However, attenuated clinical phenotype was cited in a previous article [15]. In fact, they reported a large proportion (79%) MPS type IIIB showing as the attenuated type. Attenuated phenotype is characterized by significantly slower regression of intellectual and motor abilities. A majority of patients lived well into adulthood [15]. This probably emanated from the presence of low-level residual enzyme activity that delayed storage of heparan-sulphate and hence onset and progression of symptoms. Classical MPS type IIIB is caused by mutation in NAGLU at 17q21.1 [17-18]. Missense changes p.R643C, p.S612G, p.E634K, and p.L497V were found in patients with attenuated phenotype, exclusively. Further genetic testing for such regions is ongoing. Our review contains no report of MPS at mutation related to 3q25 region. It remains unclear as to whether the clinical phenomenon of this patient was partly influenced by other etiologies such as MPS.

Clinical facial dysmorphism appearance in our present case may resemble genetic disorders such as Donohue syndrome, 3q deletion syndrome or even storage diseases [17, 19-20]; final diagnosis may depend on genetic testing. Variable expressivity and phenotype heterogeneity does exist in many genetic disorders. At present, we cannot exclude possible causal relationship between these diseases. Long-term follow-up of details on natural disease course such as neurocognitive deterioration, combined with genotype correlation, may help to predict clinical course of the disease and establish definitive diagnosis.

## Acknowledgments

This study was supported in part by grant from China Medical University Hospital (DMR-96-004).

## References

[1] Moortgat S, Verellen-Dumoulin C, Maystadt I, Parmentier B, Grisart B, Hennecker JL (2011). Developmental delay and facial dysmorphism in a child with an 8.9 Mb de novo interstitial deletion of 3q25.1-q25.32: Genotype-phenotype correlations of chromosome 3q25 deletion syndrome. Eur J Med Genet.

[2] Franceschini P, Cirillo Silengo M, Davi G, Bianco R, Biagioli M. (1983). Interstitial deletion of the long arm of chromosome 3 in a patient with mental retardation and congenital anomalies. Hum Genet.

[3] Martsolf JT, Ray M. (1983). Interstitial deletion of the long arm of chromosome 3. Ann Genet.

[4] Al-Awadi SA, Naguib KK, Farag TI, Teebi AS, Cuschieri A, Al-Othman SA (1986). Complex translocation involving chromosomes Y, 1, and 3 resulting in deletion of segment 3q23-q25. J Med Genet.

[5] Alvarado M, Bocian M, Walker AP. (1987). Interstitial deletion of the long arm of chromosome 3: case report, review, and definition of a phenotype. Am J Med Genet.

[6] Chandler KE, de Die-Smulders CE, Engelen JJ, Schrander JJ. (1997). Severe feeding problems and congenital laryngostenosis in a patient with 3q23 deletion. Eur J Pediatr.

[7] Ko WT, Lam WF, Lo FM, Chan WK, Lam TS. (2003). Wisconsin syndrome in a patient with interstitial deletion of the long arm of chromosome 3: further delineation of the phenotype. Am J Med Genet A.

[8] Rea G, McCullough S, McNerlan S, Craig B, Morrison PJ. (2010). Delineation of a recognisable phenotype of interstitial deletion 3 (q22.3q25.1) in a case with previously unreported truncus arteriosus. Eur J Med Genet.

[9] Sudha T, Dawson AJ, Prasad AN, Konkin D, de Groot GW, Prasad C. (2001). De novo interstitial long arm deletion of chromosome 3 with facial dysmorphism, Dandy-Walker variant malformation and hydrocephalus. Clin Dysmorphol.

[10] Slavotinek AM, Huson SM, Fitchett M. (1997). Interstitial deletion of band 3q25. J Med Genet.

[11] Robin NH, Magnusson M, McDonald-McGinn D, Zackai EH, Spinner NB. (1993). De novo interstitial deletion of the long arm of chromosome 3: 46,XX, del(3)(q25.1q26.1). Clin Genet.

[12] Vardarajan BN, Eran A, Jung JY, Kunkel LM, Wall DP. (2013). Haplotype structure enables prioritization of common markers and candidate genes in autism spectrum disorder. Transl Psychiatry.

[13] Dilts CV, Morris CA, Leonard CO. (1990). Hypothesis for development of a behavioral phenotype in Williams syndrome. Am J Med Genet Suppl.

[14] Shprintzen RJ, Goldberg R, Golding-Kushner KJ, Marion RW. (1992). Late-onset psychosis in the velo-cardio-facial syndrome. Am J Med Genet.

[15] Valstar MJ, Bruggenwirth HT, Olmer R, Wevers RA, Verheijen FW, Poorthuis BJ (2010). Mucopolysaccharidosis type IIIB may predominantly present with an attenuated clinical phenotype. J Inherit Metab Dis.

[16] Saini AG, Singhi P, Sahu JK, Ganesan SL, Vyas S, Rao S, et al. Hyperactivity, Unexplained Speech Delay, and Coarse Facies†Is It Sanfilippo Syndrome? J Child Neurol 2013. 10.1177/0883073813491627 23761035

[17] Yogalingam G, Hopwood JJ. (2001). Molecular genetics of mucopolysaccharidosis type IIIA and IIIB: Diagnostic, clinical, and biological implications. Hum Mutat.

[18] Zhao HG, Li HH, Bach G, Schmidtchen A, Neufeld EF. (1996). The molecular basis of Sanfilippo syndrome type B. Proc Natl Acad Sci U S A.

[19] de Bock M, Hayes I (2012). Semple R. "Donohue syndrome". J Clin Endocrinol Metab.

[20] Simovich MJ, Bland SD, Peiffer DA, Gunderson KL, Cheung SW, Yatsenko SA (2008). Delineation of the proximal 3q microdeletion syndrome. Am J Med Genet A.

